# Comparative risk of infections between JAK inhibitors versus TNF inhibitors among patients with rheumatoid arthritis: a cohort study

**DOI:** 10.1186/s13075-023-03111-w

**Published:** 2023-07-26

**Authors:** Se Rim Choi, Anna Shin, You-Jung Ha, Yun Jong Lee, Eun Bong Lee, Eun Ha Kang

**Affiliations:** 1 Division of Rheumatology, Department of Internal Medicine, Seoul National University Bundang Hospital, 166 Gumiro Bundang-gu Kyeongki-do, Seongnam-si, South Korea; 2 Department of Internal Medicine, Seoul National University College of Medicine, Seoul, South Korea; 3 Division of Rheumatology, Department of Internal Medicine, Seoul National University Hospital, Seoul, South Korea

**Keywords:** Rheumatoid arthritis, Janus kinase inhibitors, Tumor necrosis factor inhibitors, Infections, Asian

## Abstract

**Background:**

To compare infectious risk between JAK inhibitors (JAKis) versus TNF inhibitors (TNFis) among rheumatoid arthritis (RA) patients in Korea.

**Methods:**

Using 2009–2019 Korea National Health Insurance Service database, we conducted a cohort study on RA patients initiating a JAKi or TNFi. The primary outcomes were herpes zoster (HZ), serious bacterial (SBI), and opportunistic infections (OI). Propensity-score fine-stratification (PSS) and weighting were applied to adjust for > 70 baseline covariates. Hazard ratios (HRs) and 95% confidence intervals (CIs) were estimated using Cox proportional hazard models comparing JAKi versus TNFi users.

**Results:**

We included 2963 JAKi initiators PSS-weighted on 5169 TNFi initiators. During a follow-up of 1.16 years, the most frequent type of infections was HZ with incidence rate (IR) per 100 person-years of 11.54 and 4.88 in JAKi and TNFi users, respectively. The IR of SBI was 1.39 and 1.32, respectively. The OI was rare with a majority being tuberculosis and showed an IR of 0.11 and 0.49 in JAKi and TNFi users, respectively. The PSS-weighted HR (95% CI) for individual types of infections was 2.37 (2.00–2.80) for HZ, 1.04 (0.71–1.52) for SBI, and 0.25 (0.09–0.73) for OI.

**Conclusions:**

This population-based cohort study on RA patients treated with JAKi or TNFi in Korea showed an exceptionally high IR of HZ in both treatment groups compared to that from Western countries, with an approximately doubled risk associated with JAKi versus TNFi use. The risk of SBI was comparable, but the risk of OI, particularly tuberculosis, was less among JAKi than TNFi initiators.

**Supplementary Information:**

The online version contains supplementary material available at 10.1186/s13075-023-03111-w.

## Introduction

Rheumatoid arthritis (RA) is a systemic inflammatory disease in which synovial joints are the primary target of autoimmunity [1]. However, chronic inflammation causes not only the joint failure but also a wide spectrum of comorbidities [2]. Therefore, international guidelines endorse the treat-to-target strategy to achieve remission or low disease activity [3, 4]. With the introduction of biologic and targeted synthetic disease-modifying anti-rheumatic drugs (bDMARDs and tsDMARDs, respectively), clinical outcome of RA refractory to conventional DMARDs (cDMARDs) has dramatically improved [5]. In particular, Janus kinase inhibitors (JAKis) of the tsDMARD class have shown impressive efficacy against RA [6]. According to the international guidelines, the b/tsDMARDs are used as monotherapy or in combination with DMARDs including methotrexate (MTX) to treat moderate-to-severe RA [3, 4]

Infection is one of the most common treatment-emergent adverse events in RA patients due to disease-associated immune alteration and/or treatment-related immune suppression. Overall, there is a twofold risk of serious infections among RA patients compared to the non-RA population [7]. Also, there has been a particular concern for infection among RA patients treated with high efficacy DMARDs including bDMARDs or JAKis [8–10]. The incidence rate (IR) of serious infections in randomized controlled trials (RCTs) investigating tofacitinib, a JAKi, was similar to that in RCTs evaluating bDMARDs including TNF inhibitors (TNFis) in patients with RA [10]. However, unlike bacterial infections, the risk of herpes zoster (HZ) with tofacitinib was significantly higher than that with TNFis [11, 12]. In particular, higher susceptibility to developing HZ in Asian patients has been suggested [12].

Despite such backgrounds, population-based studies have been few in the real-world setting that directly compared the risk of infections of JAKis versus bDMARDs users among RA patients of Asian ancestry [13]. To meet this end, we compared the risk of HZ, serious bacterial infections (SBI), and opportunistic infections (OI) among RA patients treated with JAKis versus TNFis using the nationally representative Korea National Health Insurance Service (KNHIS) database.

## Methods

### Data source

We used the 2009–2019 KNHIS database. The KNHIS database contains longitudinal patient data including demographics, International Classification of Diseases Tenth Revision (ICD10) diagnosis codes, procedures, prescription records (drug names, prescription and dispensing dates, days’ supply, dose, and route of administration), and type of medical utilization (outpatient, inpatient, or emergency department) of all Korean citizens [14]. The Institutional Review Board of the Seoul National University Bundang Hospital approved the study protocol (X-2207–770-901) and waived the need for written patient consent based on de-identified database. This study was conducted in accordance with the principles of the Declaration of Helsinki and Good Clinical Practice Guidelines.

### Study population

RA patients with at least two ICD10 codes of RA and any DMARD (MTX, leflunomide, hydroxychloroquine, sulfasalazine, tacrolimus, cyclosporine, mizoribine, azathioprine, infliximab, adalimumab, golimumab, etanercept, abatacept, tocilizumab, rituximab, tofacitinib, or baricitinib; certolizumab not available in Korea) were eligible [15]. We also applied a V-code (V223) that indicates a copayment beneficiary (90% reduction of the cost) associated with seropositive RA in Korea. Thus, all included study participants were eventually seropositive for rheumatoid factor and/or anti-citrullinated protein antibody.

**Fig. 1:**
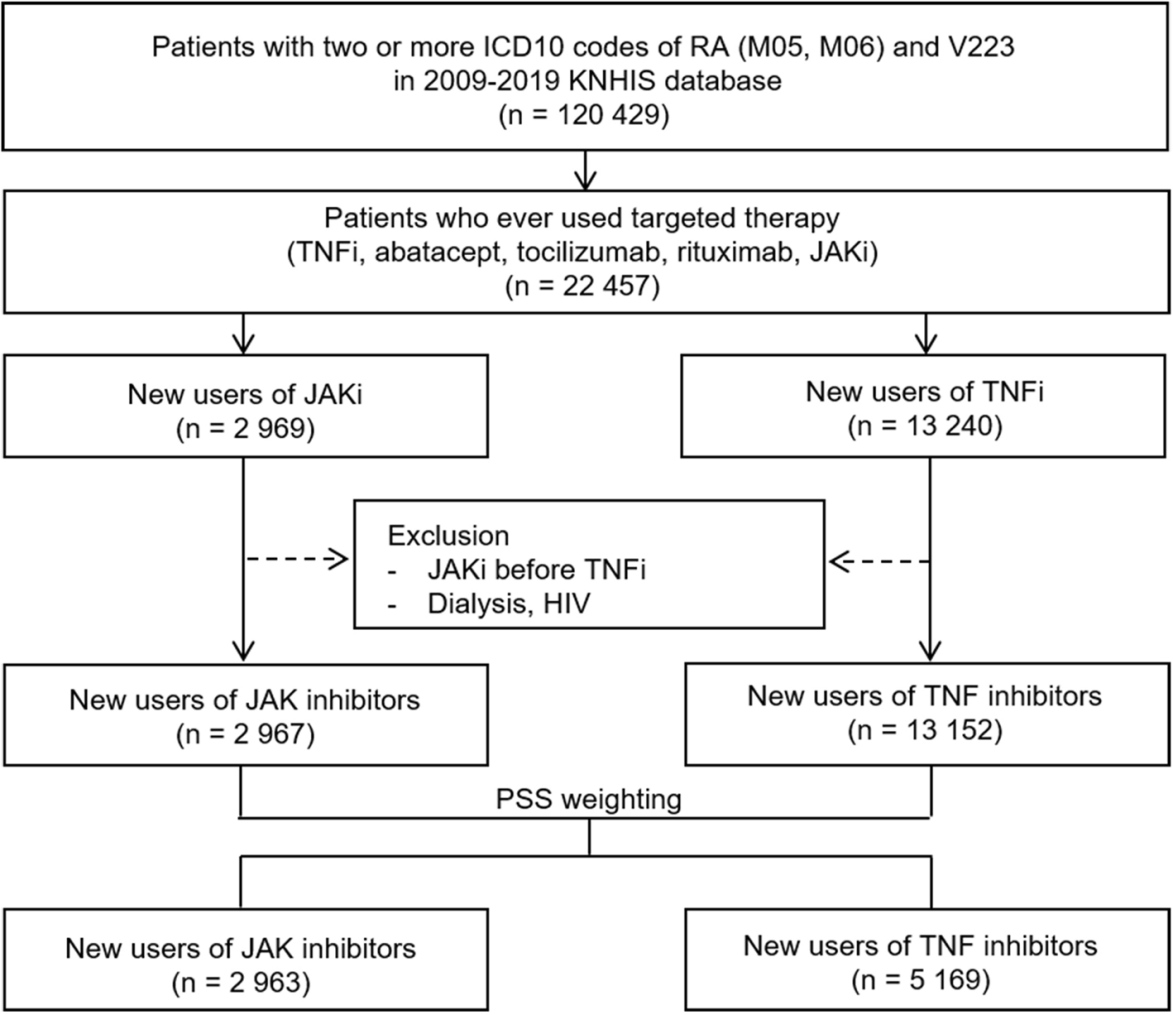
Study cohort selection process. HIV, human immunodeficiency virus; ICD, International Classification of Diseases Tenth Revision; JAKi, JAK inhibitor; KNHIS, Korea National Health Insurance Service; PSS, propensity score fine stratification; RA, rheumatoid arthritis

Among the above patients, we first selected those who initiated a JAKi (tofacitinib or baricitinib) or TNFi (infliximab, adalimumab, golimumab, or etanercept) with the first dispensing date of the corresponding index drug being defined as the index date. In Korea, bDMARD was first introduced in the market in 2004 to treat RA refractory to cDMARDs, and JAKi was first approved in 2015 as a 3rd line treatment after bDMARD failure and then approved as a 2nd line treatment after cDMARD failure from 2017. The approved dose of tofacitinib and baricitinib in Korea to treat RA does not exceed twice daily dose of 5 mg and once daily dose of 4 mg, respectively. We only included new users of individual study drugs by excluding patients who had prior use of the given study drugs during the 365-day pre-index period (= baseline period). A significant proportion of JAKi initiators used bDMARDs during the baseline period. Therefore, we allowed bDMARD use in both treatment groups as long as the bDMARDs used at baseline were not the index drug in order to utilize as many as JAKi initiators without losing comparability between the two groups (Fig. 1). But we required the TNFi group be free of JAKi during the baseline period. Other exclusion criteria included those with dialysis services or human immunodeficiency virus infection at baseline, and those hospitalized due to serious infection within 30 days prior to the index date to avoid re-hospitalization from the same episode of infection or residual effect of previous serious infection on patients’ general health.

### Outcomes

The primary outcomes were HZ, SBI, and OI. HZ cases were identified with either inpatient primary diagnosis codes or outpatient diagnosis codes plus use of anti-viral medications (i.e., acyclovir, valacyclovir, or famciclovir) within ± 7 days of the diagnosis for HZ [16]. SBI included meningitis, encephalitis, cellulitis, endocarditis/myocarditis, pneumonia, pyelonephritis, septic arthritis, osteomyelitis, and septicemia/bacteremia, and OI included tuberculosis, non-tuberculous mycobacterial infection, and systemic fungal infections (cryptococcosis, or aspergillosis). Such algorithm for SBI and OI has shown a positive predictive value of 80% using their inpatient diagnosis codes in the primary position [17].

Secondary outcomes included (1) serious HZ infection defined as the hospitalized cases where inpatient diagnosis was HZ in the primary position and (2) tuberculosis among our OI cases.

### Covariates

During the 365-day pre-index baseline period, we measured > 70 variables including patients’ demographics, index calendar years, RA medications including the number and class of bDMARD used, non-RA medications, potential risk factors for infection, use of anti-microbials, and markers of health care utilizations (listed in Table 1). We also estimated a Charlson-Deyo score for multi-morbidities [18].

**Table 1:** Baseline characteristics of study participants

	**Crude**		**PSS-weighted**		
	**JAK inhibitor**	**TNF inhibitor**	**JAK inhibitor**	**TNF inhibitor**	**SD**
	*n* = 2 967	*n* = 13 152	*n* = 2 963	*n* = 5 169	
Index age, years	55.9 ± 12.7	53.8 ± 13.5	55.8 ± 12.7	55.6 ± 13.0	0.016
Gender (%, male)	16.3	19.1	16.3	16.7	0.012
Index drug, %					
Infliximab	-	16.0	-	14.6	
Adalimumab	-	35.4	-	16.4	
Golimumab	-	15.2	-	33.3	
Etanercept	-	33.4	-	35.8	
Tofacitinib	78.8	-	78.8	-	
Baricitinib	21.2	-	21.2	-	
Index year, %					
2010		9.0			
2011		11.3			
2012		13.7			
2013		12.8			
2014		13.5			
2015	3.4	10.0	3.4	3.2	
2016	9.2	9.2	9.2	6.9	
2017	16.6	8.6	16.6	17	
2018	29.5	6.9	29.5	33	
2019	41.2	5.1	41.2	39.8	
RA medications					
Any biologics use, %	40.2	17.3	40.2	40.2	
Non-index TNFi, %	25.5	15.3	25.5	26.6	0.026
Non-TNFi biologics, %					
Abatacept	7.2	1.2	7.2	6.7	0.019
Tocilizumab	11.3	1.4	11.3	10.8	0.016
Rituximab	0.9	0.2	0.9	0.6	0.038
Number of biologics	0.5 ± 0.6	0.2 ± 0.4	0.5 ± 0.6	0.5 ± 0.6	0.002
Methotrexate, %	87.1	88.9	87.1	87.1	0.001
Leflunomide, %	39.0	45.3	39.0	39.1	0.001
Hydroxychloroquine, %	33.8	49.2	33.8	34.1	0.007
Sulfasalazine, %	23.2	35.3	23.2	24.3	0.024
Tacrolimus, %	28.2	22.0	28.1	27.0	0.025
Cyclosporine, %	0.6	2.5	0.6	0.8	0.028
Mizoribine, %	1.2	2.7	1.1	1.1	0.002
Azathioprine, %	0.5	1.2	0.5	0.4	0.016
Number of DMARDs used	2.2 ± 1.0	2.5 ± 1.1	2.2 ± 1.0	2.2 ± 1.0	0.005
NSAID, %	60.5	73.0	60.5	60.8	0.007
Cox-2 inhibitors, %	65.7	55.0	65.7	65.4	0.005
Opioids, %	13.3	25.0	13.3	12.2	0.031
Steroid use, %	95.6	96.5	95.6	95.5	0.006
Cumulative steroid dose^a^	1437 ± 1174	1641 ± 1276	1432 ± 1171	1451 ± 1191	0.015
Recent steroid use^b^, %	84.5	87.0	84.5	84.8	0.007
Recent cumulative steroid dose^a,b^	314 ± 329	395 ± 409	312 ± 310	321 ± 335	0.028
Comorbidities					
Angina, %	6.6	6.0	6.6	6.2	0.016
Myocardial infarction, %	1.4	1.3	1.4	1.5	0.013
Stroke, %	3.9	4.0	3.8	3.7	0.005
Atrial fibrillation, %	1.3	0.9	1.3	1.1	0.02
Heart failure, %	5.2	2.9	5.2	4.9	0.014
Hypertension, %	34.9	35.3	34.9	34.3	0.013
Venous thromboembolism, %	2.8	1.7	2.8	2.4	0.02
Peripheral vascular disease, %	9.9	9.1	9.9	9.7	0.005
Dyslipidemia, %	63.7	51.6	63.7	63.8	0.001
Liver disease, %	42.0	39.8	42.0	43.0	0.022
Diabetes, %	29.3	24.5	29.2	29.1	0.004
Chronic kidney disease, %	3.3	2.6	3.3	3.3	0.001
Thyroid disease, %	31.8	29.6	31.7	31.6	0.002
COPD, %	28.5	26.8	28.5	27.1	0.03
Asthma, %	15.9	14.9	15.9	14.9	0.03
Interstitial lung disease, %	5.8	3.6	5.8	4.7	0.049
Osteoporosis, %	54.6	49.6	54.6	52.9	0.034
Malignancy, %	8.0	6.8	7.9	8.5	0.02
Comorbidity index	2.6 ± 1.7	2.5 ± 1.6	2.6 ± 1.7	2.6 ± 1.7	0.012
Other medications					
ACE inhibitor or ARB, %	22.7	22.1	22.7	22.9	0.004
Beta blocker, %	11.8	15.6	11.8	11.1	0.021
Calcium channel blocker, %	23.3	21.9	23.3	23.4	0.002
Diuretic, %	14.9	23.8	14.9	14.8	0.004
Loop diuretic, %	5.4	6.8	5.4	5.4	0.002
Nitrate, %	3.0	3.0	3.0	2.4	0.041
Insulin, %	3.1	4.6	3.1	3.5	0.022
Oral hypoglycemic agent, %	9.9	10.3	9.9	10.2	0.011
Anticoagulant, %	3.0	3.0	2.9	2.8	0.011
Antiplatelet, %	8.0	9.0	8.0	7.4	0.021
Statin, %	26.2	20.5	26.2	25.9	0.008
Non-statin lipid lowering agent, %	3.9	3.4	3.9	3.8	0.005
Proton pump inhibitor, %	55.7	50.8	55.7	56.0	0.006
H2 blocker, %	50.1	63.0	50.1	49.4	0.015
Bisphosphonate, %	17.5	17.3	17.4	17.5	0.002
SERM, %	4.9	2.9	4.9	4.4	0.02
Antidepressant, %	14.9	16.0	14.9	14.9	< .001
Anti-microbials					
Use of antibiotics	71.8	73.4	71.8	71.0	0.017
Use of antivirals	10.8	8.8	10.8	11.2	0.015
Use of anti-zoster drugs	7.9	6.9	7.8	7.7	0.004
Use of antifungals, %	12.1	14.2	12.1	12.6	0.016
Healthcare use intensities					
Hospitalization, %	30.9	35.8	30.9	30.6	0.006
Number of hospitalizations	0.7 ± 1.6	0.8 ± 1.5	0.7 ± 1.6	0.7 ± 1.4	0.016
ER visit, %	15.1	18.3	15.1	14.7	0.011
Number of ER visits	0.3 ± 1.0	0.3 ± 2.8	0.2 ± 1.0	0.2 ± 1.0	0.01
Number of outpatient clinic visits	34.8 ± 27.6	35.9 ± 31.4	34.8 ± 27.6	34.6 ± 29.8	0.008
ECG ordered, %	37.9	44.8	37.8	37.9	0.002
HbA1c ordered, %	12.0	20.2	11.9	12.8	0.025
Serum creatinine test ordered, %	53.8	92.0	53.8	54.7	0.018
Lipid/cholesterol test ordered, %	50.6	86.9	50.6	51.3	0.014

Data are presented as % for binary variables and mean ± standard deviation for continuous variables

*ACE* Angiotensin-converting-enzyme, *ARB* Angiotensin receptor blocker, *COPD* Chronic obstructive pulmonary disease, *DMARD* Disease-modifying anti-rheumatic drug, *ECG* Electrocardiogram, *ER* Emergency room, *NSAID* Nonsteroidal anti-inflammatory drug, *PSS* Propensity score fine stratification, *RA* Rheumatoid arthritis, *SD* Standardized difference, *SERM* Selective estrogen receptor modulator, *TNFi* TNF inhibitors

^a^Prenisone equivalent dose

^b^Recent = within 3 months from the index date

### Statistical analysis

In our primary as-treated analysis, patients were followed from the day after the index date to the first occurrence of the following events: outcome occurrence, disenrollment, death, discontinuation of the index treatment, or adding any other DMARDs over the index treatment. Switching between different TNFis or between different JAKis was not a censoring event. Drug discontinuation was defined as no dispensing within 90 days from the expected refill date. Patients who discontinued the study medication were followed up until the last expected refill date plus 30-day grace period. The expected refill date was calculated by adding days’ supply to the last dispensing date of the study medication. The days’ supply of individual TNFi was 56 days for infliximab, 14 days for adalimumab, 28 days for golimumab, and 7 days for etanercept. For the secondary intention-to-treat (ITT) analysis, we followed patients up to 365 days after the index date without censoring on drug switching, adding, or discontinuation.

For confounding adjustment, we used propensity score (PS) fine stratification and weighting to account for > 70 baseline covariates listed in Table 1 [19]. A multivariable logistic regression model incorporated all of these covariates including the index year to estimate a PS, which was defined as the predicted probability of a patient initiating a JAKi versus TNFi given aforementioned baseline covariates. After trimming patients in the non-overlapping areas of PS, we created 50 strata based on the distribution of PS among the exposed (i.e., JAKi treatment); patients in the comparator therapy group were weighted proportionally to the distribution of the JAKi group within each of the 50 PS strata. The covariate balance between the two groups among the PS stratification (PSS) weighted study cohort was evaluated by standardized mean differences: a balanced covariate distribution was considered achieved with a standardized mean difference of < 0.1 [20]. PSS-weighted IRs of primary and secondary outcomes were calculated per 100-person-years. We used a Cox proportional hazard model estimating the hazard ratio (HR) and 95% confidence intervals (CIs). Proportional hazard assumptions were not violated in any of the models when tested using the interaction term between the exposure and follow-up time [21]. All analyses were performed using the SAS 9.4 (SAS Institute) software.

### Subgroup analysis

To investigate the risk factors associated with infections of interest, we performed analyses on subgroups stratified by age (≥ and < 60 years), concurrent use of MTX, and concurrent use of steroids. The PSS-weighting was separately done for each subgroup analysis. The interaction between treatment and individual stratifying factors was also tested using the Cox model.

## Results

### Baseline patient characteristics

We identified 2967 JAKi initiators and 13,152 TNFi initiators from the database and generated 2963 JAKi initiators (78.8% tofacitinib, 21.2% baricitinib) PSS-weighted on 5169 TNFi initiators (Fig. 1). The baseline patient characteristics before and after PSS-weighting were summarized in Table 1.

Before PSS-weighting, bDMARD use (40.2 vs 17.3%) prior to the index date was more common among JAKi than TNFi initiators, and the average number of bDMARDs used (0.5 vs 0.2) was also higher in JAKi initiators. This is expected since JAKi had been initially approved as a 3rd-line treatment after bDMARD failure in Korea and later approved as a 2nd-line treatment. TNFi than JAKi initiators showed a higher mean cumulative steroid dose (1641 vs 1437 mg of prednisone equivalent dose) and more frequently used analgesics (both non-steroidal anti-inflammatory drugs and opioids) at baseline. The comorbidity profile was comparable in general between the two groups. In particular, 7–8% of patients already experienced anti-HZ treatment before the index date. Healthcare service use was more common among TNFi initiators than JAKi: 35.8 vs. 30.9% of hospitalization, 18.3 vs. 15.1% of emergency department visits, 20.2 vs.12.0% of HbA1c test ordered, 92.0 vs 53.8% of serum creatinine test ordered, and 86.9 vs 50.6% of lipid profile test ordered.

After PSS-weighting, all of the baseline covariates were well-balanced according to a standardized difference of < 0.1. MTX use at baseline was observed in 87.1% of both treatment groups. Although MTX dose was not included in the PS-estimating logistic model, we observed a well-balanced distribution between the two groups (standardized differences of < 0.1) of mean index and maximal doses of MTX (standard deviation) during follow-up: the index dose of 9.5 (6.9) mg and maximal dose of 10.2 (6.6) mg among JAKi initiators and 9.6 (7.7) mg and 10.7 (7.2) mg among TNFi initiators.

### Comparative risk of infections between JAK inhibitor and TNF inhibitor users

The most frequent type of infections was HZ among others (Table 2). During a mean follow-up of 1.16 years, 582 cases of HZ occurred with the IR of HZ per 100 person-years of 11.54 and 4.88 in JAKi and TNFi users, respectively. The PSS-weighted HR (95% CI) of HZ was 2.37 (2.00–2.80) comparing JAKi and TNFi users. Among all HZ cases in JAKi users, 17.2% were serious, requiring hospitalizations. The risk of such serious HZ infection was even higher among JAKi users than TNFi with the PSS-weighted HR (95% CI) of 7.43 (3.91–14.11).

**Table 2:** Infectious risk comparing JAK inhibitor and TNF inhibitor users

	**JAK inhibitor** ***n* = 2 963**			**TNF inhibitor** (Ref) ***n* = 5 169**			HR (95% CI)
	Events	PY	^a^IR (95% CI)	Events	PY	^a^IR (95% CI)	
**As-treated analysis**							
Herpes zoster	361	3128	11.54 (10.47–12.72)	221	4528	4.88 (4.29–5.55)	2.37 (2.00–2.80)
Serious herpes zoster	62	3435	1.81 (1.41–2.31)	11	4682	0.24 (0.13–0.42)	7.43 (3.91–14.11)
Serious bacterial infection	48	3445	1.39 (1.05–1.85)	61	4629	1.32 (1.03–1.69)	1.04 (0.71–1.52)
Opportunistic infection	4	3489	0.11 (0.04–0.31)	23	4686	0.49 (0.33–0.74)	0.25 (0.09–0.73)
**365-day ITT**							
Herpes zoster	242	2173	11.14 (9.89–12.54)	211	4072	5.18 (4.54–5.91)	2.15 (1.79–2.59)
Serious herpes zoster	37	2263	1.64 (1.19–2.25)	12	4156	0.29 (0.16–0.51)	5.77 (3.00–11.12)
Serious bacterial infection	36	2260	1.59 (1.15–2.20)	61	4133	1.48 (1.15–1.89)	1.08 (0.72–1.63)
Opportunistic infection	3	2274	0.13 (0.04–0.41)	16	4155	0.39 (0.24–0.63)	0.35 (0.10–1.20)

*CI* Confidence interval, *HR* Hazard ratio, *IR* Incidence rate, *ITT* Intention-to-treat, *PY* Person-years

^a^Per 100 person-years

The IR of SBI was similar between the two groups: 1.39 and 1.32 per 100 person-years in JAKi and TNFi users, respectively, with the PSS-weighted HR (95% CI) of 1.04 (0.71–1.52). Upper urinary tract infection (47.7% of all SBI) was most common, followed by septic arthritis (22.0%), cellulitis (21.1%), and pneumonia (9.2%) (Supplemental Table 1).

The OI was rare with a majority (22/27, 81.5%) being tuberculosis. Four OI cases among JAKi users were tuberculosis (*n* = 2) and systemic fungal infections (*n* = 1 for systemic candidiasis, *n* = 1 for aspergillosis) while 20 out of 23 OI cases among TNFi users were tuberculosis with the rest being non-tuberculous mycobacterial infection. The PSS-weighted HR (95% CI) for OI was 0.25 (0.09–0.73) comparing JAKi versus TNFi users. The ITT results were similar.

### Subgroup analyses

We performed subgroups analyses stratified by age (≥ and < 60 years), concurrent use of MTX, and concurrent use of steroids (Supplemental Table 2). The IR of HZ, SBI, and OI was substantially higher among those aged ≥ than < 60 years. However, the combined use of MTX or steroid did not alter the IR of these infections. Although IRs of individual types of infections within each subgroup were affected by stratifying factors, the PSS-weighted HR between JAKi versus TNFi users was similar to that of the main PSS-weighted cohort (Table 3). There was no significance when interaction between treatment and stratifying factors was tested.

**Table 3:** Comparative infection risk in subgroups

	**JAK inhibitor**			**TNF inhibitor** (Ref)			HR (95% CI)
	Events	PY	^a^IR (95% CI)	Events	PY	^a^IR (95% CI)	
**Patients age < 60** years (*n* = 1 764 for JAK inhibitor vs. *n* = 3 251 for TNF inhibitor)							
Herpes zoster	178	1951	9.12 (7.93–10.49)	111	3014	3.68 (3.07–4.42)	2.47 (1.94–3.13)
Serious herpes zoster	26	2104	1.24 (0.84–1.81)	3	3095	0.10 (0.03–0.30)	12.73 (3.72–43.55)
Serious bacterial infection	27	2094	1.29 (0.89–1.88)	29	3064	0.95 (0.66–1.36)	1.41 (0.83–2.38)
Opportunistic infection	2	2126	0.09 (0.02–0.38)	3	3097	0.10 (0.03–0.30)	0.91 (0.16–5.31)
**Patients age ≥ 60 years** (*n* = 1 192 for JAK inhibitor vs. *n* = 1 798 for TNF inhibitor)							
Herpes zoster	179	1172	15.28 (13.35–17.48)	94	1430	6.57 (5.41–7.99)	2.35 (1.83–3.03)
Serious herpes zoster	35	1325	2.64 (1.91–3.66)	7	1492	0.47 (0.22–0.98)	5.57 (2.46–12.62)
Serious bacterial infection	20	1343	1.49 (0.96–2.30)	30	1475	2.03 (1.43–2.90)	0.69 (0.38–1.23)
Opportunistic infection	2	1355	0.15 (0.04–0.59)	12	1495	0.80 (0.46–1.41)	0.20 (0.04–0.88)
**No MTX combination** (*n* = 729 for JAK inhibitor vs. *n* = 1 147 for TNF inhibitor)							
Herpes zoster	81	809	10.02 (8.15–12.31)	61	1046	5.83 (4.57–7.44)	1.76 (1.26–2.46)
Serious herpes zoster	15	874	1.72 (1.04–2.83)	3	1137	0.26 (0.09–0.82)	6.94 (1.91–25.22)
Serious bacterial infection	16	875	1.83 (1.13–2.97)	20	1101	1.82 (1.18–2.80)	0.99 (0.51–1.93)
Opportunistic infection	2	891	0.23 (0.06–0.90)	8	1137	0.70 (0.35–1.40)	0.34 (0.07–1.63)
**MTX combination** (*n* = 2 221 for JAK inhibitor vs. *n* = 4 030 for TNF inhibitor)							
Herpes zoster	278	2297	12.10 (10.93–13.62)	179	3530	5.07 (4.40–5.85)	2.41 (1.99–2.91)
Serious herpes zoster	47	2535	1.85 (1.40–2.46)	8	3646	0.22 (0.11–0.44)	7.97 (3.80–16.70)
Serious bacterial infection	32	2545	1.23 (0.89–1.77)	38	3625	1.05 (0.76–1.44)	1.19 (0.74–1.92)
Opportunistic infection	2	2573	0.08 (0.02–0.31)	9	3651	0.25 (0.13–0.47)	0.33 (0.07–1.53)
**No steroid combination** (*n* = 652 for JAK inhibitor vs. *n* = 1 405 for TNF inhibitor)							
Herpes zoster	81	675	11.99 (9.78–14.71)	43	1226	3.51 (2.61–4.70)	3.42 (2.36–4.95)
Serious herpes zoster	8	742	1.08 (0.54–2.15)	1	1261	0.08 (0.01–0.56)	22.96 (1.61–327.06)
Serious bacterial infection	10	744	1.34 (0.73–2.49)	12	1251	0.96 (0.55–1.68)	1.45 (0.62–3.40)
Opportunistic infection	1	750	0.13 (0.02–0.95)	4	1260	0.32 (0.12–0.84)	0.42 (0.05–3.74)
**Steroid combination** (*n* = 2 275 for JAK inhibitor vs. *n* = 3 760 for TNF inhibitor)							
Herpes zoster	270	2419	11.16 (9.97–12.49)	166	3316	5.01 (4.32–5.81)	2.23 (1.83–2.71)
Serious herpes zoster	53	2655	2.00 (1.53–2.61)	10	3426	0.29 (0.16–0.54)	6.44 (3.30–12.57)
Serious bacterial infection	37	2661	1.39 (1.01–1.91)	38	3390	1.12 (0.82–1.54)	1.27 (0.80–2.01)
Opportunistic infection	3	2699	0.11 (0.04–0.34)	16	3430	0.47 (0.29–0.76)	0.26 (0.08–0.91)

^a^Per 100 person-years. *CI* Confidence interval, *HR* Hazard ratio, *IR* Incidence rate, *MTX* Methotrexate, *PY* Person-years

## Discussion

In this population-based cohort study, we confirmed that the IR of HZ is exceptionally high among patients with RA in Korea compared to patients from Western countries [12, 22, 23]. The risk of HZ was 2.37-fold higher in JAKi users than TNFi in our study but the IR of HZ was substantially high even in our TNFi users. The risk of SBI was comparable between the two treatments. The risk of opportunistic infection, with the majority of which cases being tuberculosis, was lower among JAKi users than TNFi.

An approximately doubled risk of HZ among JAKi users than TNFi has been shown in the ORAL Surveillance study or US claims/registry studies [22–25]. Despite the similar comparative risk of HZ between the two treatments, the IR of HZ in our study was exceptionally high among both JAKi and TNFi users, compared to that from the Western countries [26, 27]: in a study by Winthrop et al. [27] on 6192 RA patients treated with tofacitinib for a median of 3.4 years from the global clinical trials, the IR of HZ was 3.9 (95% CI 3.6 to 4.2). Yet, the IRs of HZ varied across regions, from 2.4 (95% CI 2.0–2.9) in Eastern Europe to 8.0 (95% CI 6.6–9.6) in Japan and 8.4 (95% CI 6.4–10.9) in Korea. In our real-world setting, the IR of HZ exerted by JAKi was far higher (11.54, 95% CI 10.47–12.72). Moreover, the IR of TNFi users in our study was as high as 4.88 (95% CI 4.29–5.55), also higher than the IR of patients from the Western countries treated with a JAKi [26, 27]. Surprisingly, the risk of HZ was not elevated in the Chinese or Taiwanese compared to that from a pooled analysis on the global trials [28, 29]. This finding suggests that heterogeneity exists even within Asian ethnicities. Of note, the IR (95% CI) of serious or hospitalized HZ was 1.81 (1.42–2.31) among JAKi users, which is 7.43 times than among TNFi users (IR 0.24, 95% CI 0.13–0.42).

According to the ORAL Surveillance trial [22] and the US-based registry study [25], there was no difference in the risk of serious infections comparing JAKi versus TNFi or bDMARD users. Similarly, we did not find any difference in the risk of SBI between JAKi users and TNFi. Also, pneumonia, cellulitis, and urinary tract infections were most common types of SBI as in other studies [22, 25].

The IR of OI was low as in previous RCTs. The pooled analysis of the global clinical trials and long-term extension studies showed that the IR per 100 person-years was 0.3 for OI other than tuberculosis and 0.2 for tuberculosis [26]. In our study, the IR of OI including tuberculosis was 0.11–0.49 per 100 person-years, with the majority of OI being tuberculosis. Because the case definition of OI in our study required hospitalization, our findings suggest that TNFi might confer a higher risk of severe tuberculosis than JAKi. However, this interpretation needs cautions due to the rarity of cases and further research is needed with a larger cohort of patients.

Old age, Asian ethnicity, and/or steroid use were found to be baseline risk factors for HZ in previous studies [13, 24, 27]. We found in our subgroup analyses that old age increased the risk of HZ not only among JAKi but also in TNFi users and that it was a risk factor for non-HZ infections as well. However, we did not find any significant increase in the IR of infections associated with concomitant use of steroid unlike studies on the Western populations [24, 27]. Such lack of association is similar to the Japanese multicenter study [13]. Concomitant use of MTX was not associated with a higher risk of infection, as in previous studies [13, 24, 27]. Overall, the comparative risk of infections associated with JAKi versus TNFi use was consistent across in all subgroups.

The strengths of this study are as follows: first, we used rigorous pharmacoepidemiologic methods to reduce confounding between comparator groups. The new user design with active comparator is a powerful tool to cope with both measured and unmeasured confounding [30]. In addition to that, we used PSS-based weighting to further adjust for > 70 covariates at baseline. Second, this is one of the few studies that provide a head-to-head comparison on infections between JAKis and TNFis in the real-world setting, and to the best of our knowledge, the first population-based study on Asians. Third, we used a nationally representative data to ensure high generalizability. Fourth, we used validated algorithms to define outcomes and investigated different types of infections ranging from viral, bacterial to opportunistic [16, 17]. Fourth, we performed relevant subgroup analyses to identify high risk subsets.

There are also limitations. First, inherent to any observational studies, there is a concern for residual or unmeasured confounding particularly due to lack of direct information on RA duration or activity at baseline. Nonetheless, to minimize such limitation, we used the active comparator design and further accounted for many proxies for RA activity such as the number of DMARD used, individual DMARDs used, and steroids use and their cumulative dose. Second, the number of patients receiving JAKi was small even in this nation-wide database, leading to limited power for rare outcomes. The incidence of OI was small, and more research is needed to study the comparative risk of OI to compare JAKi and TNFi. Also, the small number of outcomes in certain subgroup analyses resulted in lack of precision and wide 95% CIs. Third, the KNHIS data do not provide information on vaccination status for herpes zoster since vaccines are dispensed at individuals’ own expense.

## Conclusion

Taken together, despite the similar comparative risk of HZ among JAKi users versus TNFi to that in other ethnic groups, the IR of HZ was far higher among RA patients in Korea. We were able to confirm that the risk of SBI was very similar between the two treatments. We also cautiously suggest that TNFi might confer a higher risk for tuberculosis that requires hospitalizations. When we performed a subgroup analysis to identify risk factors associated with infections in JAKi and TNFi users, we found that the elderly was more susceptible for all types of infections but concomitant use of either steroid or MTX did not increase the risk of infections.

## Supplementary Information

**Additional file 1:** **Supplemental Table 1. **Site of serious bacterial infections. **Supplemental Table 2. **Baseline characteristics of PSS-weighted subgroups. 

## Data Availability

Data are available upon reasonable request.

## Abbreviations

bDMARD: Biologic disease-modifying anti-rheumatic drug
cDMARD: Conventional disease-modifying anti-rheumatic drug
CI: Confidence interval
HR: Hazard ratio
HZ: Herpes zoster
IR: Incidence rate
JAKi: Janus kinase inhibitor
KNHIS: Korea National Health Insurance Service
MTX: Methotrexate
OI: Opportunistic infection
PSS: Propensity-score fine stratification
RA: Rheumatoid arthritis
SBI: Serious bacterial infection
TNFi: Tumor necrosis factor inhibitor
tsDMARD: Targeted synthetic disease-modifying anti-rheumatic drug
