# Supplementary material for: Comparative risk of infections between JAK inhibitors versus TNF inhibitors among patients with rheumatoid arthritis: a cohort study
Source: Arthritis Res Ther. 2023 Jul 26;25:129. doi: 10.1186/s13075-023-03111-w (PMC10369724; doi:10.1186/s13075-023-03111-w)
Supplement: Supplementary file 1 — Additional file 1: Supplemental Table 1. Site of serious bacterial infections. Supplemental Table 2. Baseline characteristics of PSS-weighted subgroups. [file 13075_2023_3111_MOESM1_ESM.docx]

**Supplemental Table 1.** Site of serious bacterial infections

|  | **JAK inhibitor** (n = 2 963) | | **TNF inhibitor** (n = 5 169) | |
| --- | --- | --- | --- | --- |
| Site of infections | No. of events | ^a^IR (95% CI) | Events | ^a^IR (95% CI) |
| All serious bacterial infections | 48 | 1.39 (1.05-1.85) | 61 | 1.32 (1.03-1.69) |
| Pyelonephritis | 26 | 0.75 (0.51-1.10) | 26 | 0.56 (0.38-0.82) |
| Pneumonia | 5 | 0.14 (0.06-0.34) | 5 | 0.11 (0.04-0.26) |
| Cellulitis | 7 | 0.20 (0.10-0.42) | 16 | 0.34 (0.21-0.56) |
| Septic arthritis | 5 | 0.14 (0.06-0.34) | 19 | 0.41 (0.26-0.64) |
| Bacteremia | 4 | 0.12 (0.04-0.31) | 7 | 0.15 (0.07-0.31) |
| Osteomyelitis | 0 | 0 | 0 | 0 |
| Meningitis | 0 | 0 | 0 | 0 |
| Endocarditis | 1 | 0.03 (0.004-0.20) | 0 | 0 |
| Encephalitis | 0 | 0 | 0 | 0 |

^a^per 100 person-years

CI = confidence interval, IR = incidence rate

**Supplemental Table 2.** Baseline characteristics of PSS-weighted subgroups

|  | **age <60** | | **Age≥60 years** | | **No MTX**  **combination** | | **MTX**  **combination** | | **No steroid combination** | | **Steroid**  **combination** | |
| --- | --- | --- | --- | --- | --- | --- | --- | --- | --- | --- | --- | --- |
|  | **JAKi** | **TNFi** | **JAKi** | **TNFi** | **JAKi** | **TNFi** | **JAKi** | **TNFi** | **JAKi** | **TNFi** | **JAKi** | **TNFi** |
|  | N=1764 | N=3251 | N=1192 | N=1798 | N=729 | N=1147 | N=2221 | N=4030 | N=652 | N=1405 | N=2275 | N=3760 |
| Index age, years | 47.7±9.1 | 47.7±9.3 | 67.9±6.0 | 67.6±5.8 | 57.9 ±13.4 | 58.3 ±12.1 | 55.2±12.4 | 55.0±12.9 | 56.8±12.2 | 56.6±11.9 | 55.5±12.9 | 55.1±13.2 |
| Gender (%, male) | 12.9 | 13.9 | 21.3 | 20.5 | 17.3 | 18.2 | 15.9 | 16.1 | 15.5 | 17.3 | 16.6 | 16.7 |
| Index year (%) |  |  |  |  |  |  |  |  |  |  |  |  |
| 2015 | 3.4 | 3.1 | 3.5 | 3.0 | 4.7 | 4.1 | 2.9 | 2.8 | 2.8 | 2.3 | 3.6 | 3.3 |
| 2016 | 9.2 | 6.5 | 9.4 | 7.5 | 12.2 | 11.4 | 8.2 | 5.4 | 8.9 | 7.2 | 9.4 | 6.5 |
| 2017 | 17.7 | 19.5 | 14.9 | 14.7 | 17.6 | 20.5 | 16.3 | 17.4 | 17.0 | 17.7 | 16.6 | 18.3 |
| 2018 | 31.0 | 34.3 | 27.3 | 31.3 | 28.4 | 28.1 | 30.0 | 34.2 | 29.0 | 31.9 | 29.6 | 33.0 |
| 2019 | 38.7 | 36.7 | 44.8 | 43.6 | 37.2 | 36.0 | 42.6 | 40.2 | 42.3 | 41.0 | 40.8 | 39.0 |
| RA medications |  |  |  |  |  |  |  |  |  |  |  |  |
| Non-index TNFi, % | 26.5 | 28.7 | 23.9 | 24.8 | 25.2 | 27.3 | 26.3 | 26.9 | 25.6 | 27.8 | 26.0 | 27.2 |
| Abatacept | 6.0 | 6.0 | 8.8 | 7.6 | 11.4 | 10.5 | 5.8 | 5.7 | 8.0 | 6.3 | 6.7 | 6.9 |
| Tocilizumab | 11.1 | 10.4 | 11.4 | 11.5 | 17.8 | 15.4 | 9.1 | 8.7 | 8.9 | 6.9 | 12.0 | 11.2 |
| Rituximab | 0.8 | 0.4 | 0.8 | 0.7 | 0.0 | 0.0 | 0.8 | 0.6 | 0.0 | 0.0 | 1.0 | 0.4 |
| Number of biologics | 0.5±0.6 | 0.5±0.7 | 0.5±0.6 | 0.5±0.7 | 0.5±0.6 | 0.6±0.7 | 0.4±0.6 | 0.4±0.6 | 0.4±0.6 | 0.4±0.6 | 0.5±0.6 | 0.5±0.7 |
| Methotrexate, % | 89.6 | 89.2 | 83.4 | 82.7 | 51.0 | 48.3 | 99.2 | 99.0 | 85.4 | 86.4 | 87.7 | 87.5 |
| Leflunomide, % | 38.7 | 36.8 | 39.6 | 41.3 | 42.7 | 38.9 | 37.9 | 37.9 | 39.7 | 38.9 | 38.8 | 38.4 |
| Hydroxychloroquine, % | 33.8 | 33.8 | 34.0 | 35.7 | 34.0 | 32.0 | 33.9 | 33.7 | 34.4 | 37.8 | 33.8 | 34.6 |
| Sulfasalazine, % | 23.7 | 23.4 | 22.6 | 23.6 | 25.9 | 25.8 | 22.3 | 22.9 | 21.9 | 21.7 | 23.8 | 24.2 |
| Tacrolimus, % | 29.9 | 28.9 | 25.7 | 23.8 | 34.0 | 33.5 | 26.4 | 26.2 | 28.2 | 27.4 | 28.0 | 28.0 |
| Cyclosporine, % | 0.8 | 1.0 | 0.3 | 0.4 | 1.5 | 2.8 | 0.3 | 0.3 | 0.6 | 3.4 | 0.6 | 0.6 |
| Mizoribine, % | 1.1 | 1.1 | 1.2 | 1.4 | 2.3 | 2.4 | 0.8 | 0.7 | 1.8 | 2.9 | 1.0 | 1.1 |
| Mycophenolate, % | 0.3 | 0.2 | 0.4 | 0.6 | 0.7 | 0.4 | 0.3 | 0.2 | 0.8 | 0.7 | 0.3 | 0.2 |
| Bucillamine, % | 3.0 | 3.5 | 2.0 | 2.1 | 3.4 | 3.1 | 2.3 | 2.6 | 2.6 | 4.5 | 2.6 | 2.6 |
| Azathioprine, % | 0.5 | 0.4 | 0.5 | 0.3 | 0.8 | 0.8 | 0.4 | 0.3 | 0.2 | 0.1 | 0.6 | 0.5 |
| Number of DMARDs used | 2.2±1.0 | 2.2±1.0 | 2.1±1.0 | 2.1±1.1 | 2.0±1.2 | 1.9±1.2 | 2.2±1.0 | 2.2±1.0 | 2.2±1.0 | 2.2±0.9 | 2.2±1.0 | 2.2±1.0 |
| NSAID, % | 59.2 | 59.9 | 62.6 | 61.2 | 60.6 | 64.6 | 60.6 | 60.4 | 55.7 | 56.8 | 61.8 | 61.0 |
| Cox-2 inhibitors, % | 61.8 | 59.9 | 71.6 | 72.4 | 65.0 | 66.6 | 65.9 | 65.5 | 63.8 | 65.8 | 66.3 | 65.6 |
| Opioids, % | 9.8 | 8.4 | 18.5 | 17.4 | 18.4 | 20.5 | 11.5 | 9.7 | 12.1 | 12.4 | 13.5 | 12.7 |
| Steroid use, % | 95.7 | 95.5 | 95.4 | 95.6 | 93.7 | 95.8 | 96.2 | 96.0 | 83.7 | 85.9 | 99.4 | 99.3 |
| Cumulative steroid dose^a^ | 1396±1077 | 1394±1167 | 1489 ±1301 | 1526 ±1240 | 1480±1214 | 1498 ±1390 | 1415 ±1155 | 1419 ±1137 | 606±837 | 639±795 | 1685±1143 | 1705±1173 |
| Recent Steroid use^b^, % | 84.5 | 84.1 | 84.5 | 85.9 | 82.3 | 83.2 | 85.2 | 84.6 | 51.2 | 54.8 | 94.5 | 94.8 |
| Recent cumulative steroid dose^a,b^ | 303±286 | 309±321 | 331±383 | 350±382 | 339±337 | 343±466 | 303±299 | 306±298 | 127±206 | 137±238 | 374±310 | 386±355 |
| Comorbidities |  |  |  |  |  |  |  |  |  |  |  |  |
| Angina, % | 2.7 | 2.5 | 12.4 | 10.6 | 8.4 | 8.1 | 5.9 | 5.0 | 6.3 | 6.6 | 6.6 | 6.2 |
| Myocardial infarction, % | 0.7 | 0.7 | 2.3 | 2.3 | 1.2 | 1.6 | 1.4 | 1.2 | 0.5 | 0.4 | 1.6 | 1.7 |
| Stroke, % | 2.0 | 2.1 | 6.7 | 5.8 | 4.8 | 5.3 | 3.5 | 3.2 | 2.9 | 2.6 | 4.2 | 4.1 |
| Atrial fibrillation, % | 0.4 | 0.2 | 2.5 | 1.8 | 1.8 | 0.8 | 1.1 | 0.9 | 1.4 | 1.1 | 1.3 | 0.8 |
| Heart Failure, % | 2.4 | 2.4 | 9.2 | 7.9 | 8.4 | 8.4 | 4.1 | 3.4 | 6.6 | 5.2 | 4.7 | 4.1 |
| Hypertension, % | 21.4 | 20.4 | 54.9 | 53.6 | 41.6 | 40.7 | 32.5 | 31.3 | 38.3 | 37.2 | 33.8 | 32.5 |
| Venous thromboembolism, % | 2.0 | 1.9 | 3.9 | 3.5 | 3.3 | 3.9 | 2.6 | 2.1 | 2.5 | 1.7 | 2.9 | 2.7 |
| Peripheral vascular disease, % | 6.3 | 5.8 | 15.2 | 15.4 | 10.4 | 10.3 | 9.6 | 9.1 | 10.1 | 10.0 | 9.8 | 9.2 |
| Dyslipidemia, % | 57.7 | 57.2 | 72.8 | 73.0 | 64.1 | 60.7 | 63.6 | 62.8 | 64.1 | 61.5 | 63.6 | 63.2 |
| Liver disease, % | 40.5 | 41.7 | 44.2 | 45.2 | 39.6 | 42.4 | 42.7 | 43.3 | 38.3 | 37.9 | 43.3 | 43.9 |
| Diabetes, % | 21.3 | 21.8 | 41.1 | 41.1 | 30.3 | 30.8 | 28.8 | 27.2 | 28.7 | 27.7 | 29.5 | 28.7 |
| Chronic kidney disease, % | 1.4 | 1.3 | 6.1 | 6.7 | 5.9 | 7.6 | 2.3 | 2.0 | 4.1 | 3.2 | 3.0 | 3.0 |
| Thyroid disease, % | 30.1 | 30.1 | 34.2 | 32.4 | 28.4 | 26.9 | 32.8 | 31.6 | 28.4 | 31.9 | 32.8 | 31.8 |
| COPD, % | 21.3 | 19.4 | 39.2 | 39.1 | 32.2 | 28.8 | 27.2 | 25.2 | 28.8 | 26.6 | 28.3 | 25.6 |
| Asthma, % | 11.6 | 11.3 | 22.1 | 20.2 | 16.7 | 16.0 | 15.7 | 14.5 | 15.0 | 12.1 | 16.1 | 15.0 |
| Interstitial lung disease, % | 3.3 | 2.7 | 9.6 | 7.6 | 10.3 | 9.8 | 4.3 | 2.9 | 5.1 | 3.1 | 6.0 | 5.1 |
| Osteoporosis, % | 44.7 | 42.6 | 69.3 | 70.1 | 57.9 | 58.7 | 53.6 | 52.5 | 49.2 | 47.3 | 56.1 | 54.0 |
| Malignancy, % | 5.9 | 6.6 | 11.1 | 11.0 | 9.9 | 11.8 | 7.3 | 7.3 | 7.7 | 7.6 | 8.0 | 8.5 |
| Comorbidity index | 2.2±1.3 | 2.1±1.4 | 3.2±1.9 | 3.2±1.9 | 2.8±1.9 | 2.9±1.9 | 2.5±1.6 | 2.4±1.5 | 2.6±1.7 | 2.5±1.6 | 2.6±1.7 | 2.5±1.7 |
| Other medications |  |  |  |  |  |  |  |  |  |  |  |  |
| ACE inhibitor or ARB, % | 13.1 | 13.3 | 36.9 | 37.4 | 28.3 | 29.6 | 20.7 | 20.3 | 24.5 | 24.2 | 22.1 | 21.9 |
| Beta blocker, % | 7.4 | 6.6 | 18.3 | 17.4 | 16.5 | 15.6 | 10.2 | 9.0 | 13.0 | 12.3 | 11.4 | 10.4 |
| Calcium channel blocker, % | 13.4 | 13.7 | 38.0 | 37.6 | 29.2 | 29.6 | 21.3 | 21.3 | 24.1 | 23.7 | 22.8 | 22.7 |
| Diuretic, % | 9.8 | 9.9 | 22.4 | 22.0 | 18.9 | 18.7 | 13.5 | 12.9 | 14.6 | 14.0 | 14.8 | 14.1 |
| Loop diuretic, % | 3.5 | 3.3 | 8.2 | 7.8 | 9.5 | 9.0 | 4.0 | 3.8 | 7.2 | 7.5 | 4.7 | 4.5 |
| Nitrate, % | 1.1 | 0.7 | 6.0 | 4.6 | 4.5 | 3.0 | 2.5 | 2.0 | 2.8 | 2.8 | 3.0 | 2.3 |
| Insulin, % | 1.3 | 1.1 | 5.9 | 6.9 | 5.9 | 7.1 | 2.3 | 2.2 | 4.9 | 7.1 | 2.5 | 2.6 |
| Oral hypoglycemic agent, % | 5.4 | 6.1 | 16.5 | 16.6 | 13.4 | 13.9 | 8.7 | 8.7 | 12.9 | 15.0 | 9.1 | 8.8 |
| Anticoagulant, % | 1.2 | 0.9 | 5.5 | 4.5 | 4.7 | 3.7 | 2.4 | 1.9 | 2.8 | 2.3 | 3.0 | 2.4 |
| Antiplatelet, % | 3.3 | 2.7 | 15.0 | 13.8 | 9.6 | 8.4 | 7.4 | 6.3 | 9.5 | 8.5 | 7.5 | 7.1 |
| Statin, % | 17.8 | 18.0 | 38.7 | 37.3 | 28.0 | 25.9 | 25.6 | 25.4 | 31.7 | 28.3 | 24.6 | 24.9 |
| Non-statin lipid lowering agent, % | 2.8 | 2.9 | 5.6 | 5.0 | 5.1 | 4.9 | 3.5 | 3.6 | 4.4 | 4.9 | 3.8 | 3.5 |
| Proton pump inhibitor, % | 48.9 | 50.4 | 65.8 | 66.0 | 59.3 | 57.8 | 54.5 | 55.0 | 51.4 | 50.9 | 56.9 | 56.3 |
| H2 blocker, % | 45.8 | 43.8 | 56.5 | 54.4 | 51.4 | 50.1 | 49.7 | 49.1 | 48.6 | 48.2 | 50.5 | 49.7 |
| Bisphosphonate, % | 8.8 | 8.5 | 30.2 | 31.1 | 20.3 | 19.7 | 16.5 | 16.5 | 15.6 | 14.7 | 18.0 | 17.5 |
| SERM, % | 2.3 | 2.1 | 8.7 | 8.7 | 6.4 | 5.3 | 4.3 | 4.2 | 4.6 | 4.7 | 5.0 | 4.6 |
| Antidepressant, % | 11.3 | 10.5 | 20.2 | 20.5 | 17.6 | 18.8 | 14.1 | 13.8 | 15.3 | 14.8 | 14.9 | 14.0 |
| Anti-microbials |  |  |  |  |  |  |  |  |  |  |  |  |
| Use of antibiotics | 68.1 | 66.9 | 77.1 | 77.2 | 73.3 | 73.1 | 71.4 | 69.6 | 69.3 | 73.5 | 72.4 | 70.8 |
| Use of antivirals | 10.3 | 10.5 | 11.6 | 11.2 | 12.2 | 12.4 | 10.3 | 10.1 | 11.0 | 10.4 | 10.9 | 10.8 |
| Use of anti-zoster drugs | 7.4 | 6.5 | 8.6 | 9.0 | 8.2 | 6.9 | 7.7 | 7.4 | 8.7 | 7.8 | 7.7 | 7.1 |
| Use of antifungals, % | 12.4 | 12.0 | 11.7 | 13.2 | 13.6 | 15.4 | 11.6 | 12.4 | 11.0 | 12.6 | 12.5 | 13.5 |
| Healthcare use intensities |  |  |  |  |  |  |  |  |  |  |  |  |
| Hospitalization, % | 24.1 | 24.4 | 40.9 | 40.6 | 37.7 | 40.5 | 28.5 | 27.1 | 27.3 | 29.0 | 32.0 | 30.3 |
| Number of hospitalizations | 0.5±1.3 | 0.5±1.2 | 1.0±1.9 | 0.9±1.7 | 0.9±1.8 | 0.9±1.6 | 0.6±1.5 | 0.6±1.3 | 0.6±1.3 | 0.7±1.6 | 0.7±1.7 | 0.6±1.3 |
| ER visit, % | 12.8 | 12.5 | 18.5 | 17.4 | 18.2 | 15.4 | 13.9 | 14.4 | 13.8 | 12.2 | 15.5 | 14.8 |
| Number of ER visits | 0.2±0.9 | 0.2±0.9 | 0.3±1.2 | 0.3±1.0 | 0.3±1.6 | 0.2±1.0 | 0.2±0.7 | 0.2±0.9 | 0.2±1.4 | 0.2±0.7 | 0.3±0.9 | 0.2±0.9 |
| Number of outpatient clinic visits | 29±22 | 28±22 | 44±32 | 45±37 | 39±33 | 39±33 | 34±26 | 33±27 | 34±28 | 33±29 | 35±28 | 35±30 |
| ECG ordered, % | 29.4 | 30.2 | 50.3 | 49.1 | 44.3 | 47.8 | 35.6 | 34.7 | 34.0 | 36.2 | 39.2 | 37.8 |
| HbA1C ordered, % | 9.2 | 9.7 | 16.0 | 16.7 | 16.0 | 17.9 | 10.5 | 10.7 | 11.3 | 11.1 | 12.1 | 12.2 |
| Serum creatinine test ordered, % | 55.6 | 57.3 | 51.2 | 52.0 | 58.3 | 60.5 | 52.2 | 53.7 | 52.0 | 54.0 | 54.3 | 55.6 |
| Lipid profile test ordered, % | 51.9 | 53.4 | 48.7 | 50.1 | 54.9 | 57.1 | 49.1 | 50.4 | 47.9 | 49.4 | 51.4 | 52.5 |

Data are presented as % for binary variables and mean ± standard deviation for continuous variables.

^a^Prednisolone equivalent dose; ^b^Recent = within 3 months from the index date

ACE = angiotensin-converting-enzyme, ARB = angiotensin receptor blocker, COPD = chronic obstructive pulmonary disease, DMARD = disease modifying anti-rheumatic drug, ECG = electrocardiogram, ER = emergency room, JAKi = JAK inhibitors, MTX = methotrexate, NSAID = nonsteroidal anti-inflammatory drug, PSS = propensity score fine stratification, RA = rheumatoid arthritis, SD = standardized deviation, SERM = selective estrogen receptor modulator, TNFi = TNF inhibitors
